# Enamel defects in extracted and exfoliated teeth from patients with Amelogenesis Imperfecta, measured using the extended enamel defects index and image analysis

**DOI:** 10.1016/j.archoralbio.2008.07.008

**Published:** 2009-12

**Authors:** R.N. Smith, C. Elcock, A. Abdellatif, B. Bäckman, J.M. Russell, A.H. Brook

**Affiliations:** a International Collaborating Centre in Oro-facial Genetics and Development, University of Liverpool, Liverpool L69 3GN, UK; b Department of Oral Health and Development, School of Clinical Dentistry, University of Sheffield, Claremont Crescent, Sheffield S10 2TA, UK; c Department of Odontology/Paediatric Dentistry, Umeå University, Umeå, Sweden; d Department of Corporate Information and Computing Studies, University of Sheffield, Sheffield S10 3TA, UK

**Keywords:** Amelogenesis Imperfecta, Enamel defects, Image analysis, EDI

## Abstract

**Aims:**

To enhance the phenotypic description and quantification of enamel defects from a North Sweden sample of extracted and exfoliated teeth originating from families with Amelogenesis Imperfecta by use of the extended enamel defects index (EDI) and image analysis to demonstrate the comparable reliability and value of the additional measurements.

**Methods and results:**

The sample comprised 109 deciduous and 7 permanent teeth from 32 individuals of 19 families with Amelogenesis Imperfecta in Northern Sweden. A special holder for individual teeth was designed and the whole sample was examined using the extended EDI and an image analysis system. In addition to the extended EDI definitions, the calibrated images were measured for tooth surface area, defect area and percentage of surface affected using image analysis techniques. The extended EDI was assessed using weighted and unweighted Kappa statistics. The reliability of imaging and measurement was determined using Fleiss’ intra-class correlation coefficient (ICCC). Kappa values indicated good or excellent intra-operator repeatability and inter-operator reproducibility for the extended EDI. The Fleiss ICCC values indicated excellent repeatability for the image analysis measurements. Hypoplastic pits on the occlusal surfaces were the most frequent defect in this sample (82.6%). The occlusal surface displayed the most post-eruptive breakdown (39.13%) whilst the incisal portion of the buccal surfaces showed most diffuse opacities (53.4%). Image analysis methods demonstrated the largest mean hypoplastic pit areas were on the lingual surfaces. The largest mean post-eruptive breakdown areas were on the lingual surfaces of posterior teeth. The largest mean demarcated opacity areas were found on the labial surfaces.

**Conclusions:**

The extended EDI and the standardised image acquisition and analysis system provided additional information to conventional measurement techniques. Additional phenotypic variables were described.

## 1 Introduction

During the extensive period of enamel formation many genetic and environmental factors affect the developmental process and some of these factors induce defects.^1,2^ These defects may be quantitative, i.e. hypoplasia, or qualitative, i.e. opacity, or a combination of both. After eruption hypomineralised areas may undergo breakdown, e.g. by chipping. The clinical features and extent of defects can vary from a limited area on one tooth surface to widespread involvement, affecting multiple surfaces and teeth.^3^

Clinical indices have been used for recording such defects, including the Al-Alousi index,^4^ developmental defects of enamel index,^5^ tooth surface index of fluorosis,^6^ modified developmental defects of enamel index^7^ and the enamel defects index (EDI).^8,9^ The extended EDI (detailed below) provides further details of defect shape and size enhancing the value of the EDI.

Photographic methods have been developed to increase the accuracy of recording.^10–16^ Such objective approaches have significant advantages including capture of a permanent record, facilitation of a blind examination and reproducibility determination, and application of more than one recording and investigating method by researchers remote from the study site to the same material.^16^

Some *in vitro* studies have examined teeth using a magnifying glass or binocular microscope and used needlepoint callipers to obtain manual measurements of defects.^17,18^ However, enamel defects are often of irregular shape and frequently have ill-defined boundaries presenting difficulties in accurate quantification by manual techniques.^17–20^

Accurate recording, where possible, of developmental defects of enamel is essential in attempting to investigate the aetiology, and has applications in clinical dentistry, dental epidemiology and anthropology.^21^ These applications include genotype-phenotype correlations in amelogenesis imperfect where there is interest in relating different mutations in the same gene and the different modified proteins from the normal they produce to the appearance of the formed enamel. Similarly in investigating molar-incisor-hypomineralisation accurate measurement of the nature and extent of the enamel defects in different affected individuals contributes both to determining the clinical need and understanding the aetiology. Other applications are in studies of enamel defects possibly related to excess fluoride ingestion and to archaeological studies where environmental stress and toxins are being investigated.

Improved accuracy and increased reproducibility of methods are required to aid diagnosis and to permit better comparisons between studies. Image analysis techniques permit objective measurements enhancing those available from indices with comparable or increased reliability.^9,22^ Standardisation of the image acquisition process addresses some of the limitations of previous photographic and microscopic techniques and has therefore been applied for measurement of enamel defects in conjunction with the extended EDI in this study.

## 2 Aims

The aims of this study were to apply the extended EDI and digital image analysis acquisition and measurement techniques for measurement of enamel defects to enhance the quantification and description of enamel opacities, hypoplastic lesions and post-eruptive breakdown on extracted and exfoliated teeth from a group of North Swedish families with Amelogenesis Imperfecta.

## 3 Materials and methods

### 3.1 Sample

The sample comprised 109 deciduous and 7 permanent anterior and posterior teeth collected by one of the authors in Northern Sweden from 32 individuals of 19 families with Amelogenesis Imperfecta.^23^ The teeth were initially stored in alcohol, but prior to imaging, they were transferred into normal saline and thymol for 1 week. The teeth were coded and examined blindly. The whole sample was examined for presence of enamel defects using the extended EDI and an image analysis system. To provide more detail the teeth were divided visually into the cervical and incisal portions of the labial and lingual views when assessing using the extended EDI. It is acknowledged that occlusal wear would have occurred within the incisal portion but the division was still thought to provide further information regarding defect distribution. The occlusal view was assessed as one view.

### 3.2 Purpose-built tooth holder

**Fig. 1:**
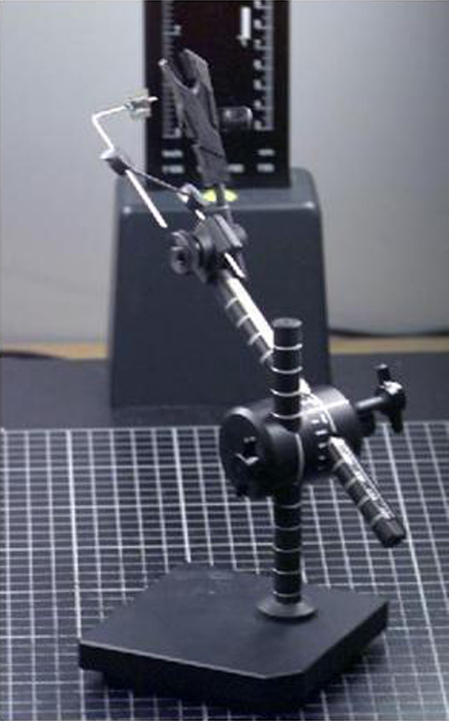
Custom built multi-axes adjustable calibrated holder for individual teeth.

A special holder for individual teeth was designed, allowing calibrated movements in all three axes of rotation and incorporated a 5-mm scale to provide linear calibration of each image (Fig. 1).

### 3.3 Image analysis system

A 32-bit digital camera Kodak Nikon DCS 410 was attached to a gridded-base copy stand (Kaiser, Germany). The stand also supported two white strip lights (RB 5000, with Phillips Fluorescent daylight bulbs, Kaiser, Germany), and two Portaflash 220 slave flashguns (Jessops) with white opaque filters. Each flashgun was covered with polarizing film, with the polarizing effect direction set the same for both flashes, and at 90° to that of a circular polarizing filter attached to the camera lens. The camera was connected to a Viglen CX1 Dual processor, Viglen, Ltd., UK) via an Adaptec 2940 SCSI card (KJP, Ltd., UK).

### 3.4 Image acquisition

After drying the teeth of any excess moisture with a soft tissue, they were positioned with the camera lens parallel to the tooth surface. The position of the camera, tooth holder and illumination was adjusted as necessary and recorded. The shutter speed and camera aperture were set and also recorded. Each tooth surface (occlusal, buccal, lingual, and proximal) was imaged under both flash and strip light illumination. Images were acquired using Adobe Photoshop (V5.02, Adobe Systems, Ltd., Europe) and viewed on the computer screen. Each image was checked for quality and re-taken if necessary by each of the two examiners. The images were saved as tagged image format files (Tiffs). A permanent database was created with images stored on 1.7 GB optical disks (Hitachi OD152 Optical Drive, CNT Direct, UK).

The first image for each surface was captured with both strip lights on. Two subsequent images were captured using one each of the two strip lights in turn. This provided images illuminated from opposite sides so that the whole tooth surface could be fully examined for any hypoplastic defect requiring shadows to fully define. Finally, the tooth was imaged using polarised flashlights to give clear images of enamel opacities without reflections. All four image types could be acquired within a matter of seconds.

### 3.5 Enamel defects index

The enamel defects index was used in this study. The scoring was done by two examiners on the same digital images as for image analysis measurement. This method involves the binary scoring of three defect categories (present or absent) and include enamel hypoplasia, opacity and post-eruptive breakdown having eight possible scores (000 to 111) per tooth surface.^2,8^ The index was expanded with sub categories for hypoplasia, opacity and post-eruptive breakdown:

• *Hypoplasia*:
  ∘ *Pits*: tiny areas of enamel loss, which could be single, multiple, shallow or deep, scattered or in rows.
  ∘ *Groove*: single or multiple, narrow or wide (maximum 2 mm) grooves of enamel loss.
  ∘ *Area*: partial or complete absence or enamel over a considerable area of the tooth.
• *Opacity*:
  ∘ *Demarcated opacity*: characterised by a distinct and clear boundary with the adjacent normal enamel. They can be white, cream or brown in colour.
  ∘ *Diffuse opacity*: can have a linear, patchy or patchy confluent form and no clear boundary with the surrounding normal enamel.
• *Post-eruptive breakdown*:
  Includes the loss of enamel surface after eruption and is characterised by sharp and irregular borders. These subtypes are also scored as present or absent (0 or 1).^9^

### 3.6 Analysis of images

**Fig. 2:**
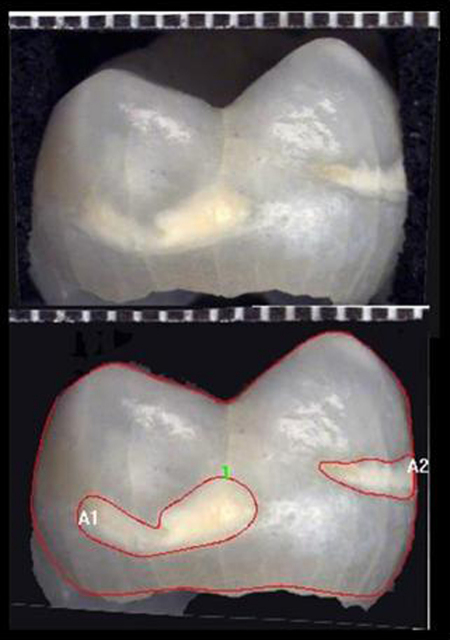
Deciduous molar showing total view surface area and demarcated opacities outlined.

Image Pro-Plus (V. 4.0, Media Cybernetics, USA) was used for analysis of areas by image analysis. The calibrated images were measured manually for tooth surface area, defect area and percentage of tooth surface affected by the defect (Fig. 2).

### 3.7 Reliability

#### 3.7.1 EDI

The error in scoring each tooth surface was quantified by unweighted Kappa^24^ and weighted kappa.^25,26^ Kappa values were calculated to assess intra- and inter-operator reliability of the extended EDI scoring by two examiners. A weakness of the standard Kappa statistic is that it takes no account of the degree of disagreement as all disagreements are treated equally. Often it is preferable to give different weights to different disagreements. To reflect the number of correctly matched digits between the two examiners, numerical weights were used. The Kappa coefficients were divided into categories as described by Landis and Kock.^25^ Measurements were made from the buccal and lingual surfaces and all scores were assessed together.

Logistic regression was applied to the EDI scoring as the main data depend on the presence or absence of the defect.^27^

Sokal and Sneath^28^ similarity measure was computed to define the most common defined defects. This is a similarity measure for binary data that yields the conditional probability that a characteristic of one item is in the presence or absence of the characteristic of another item.

All statistics were calculated using the SPSS software package (V. 10.0, Statistical Solutions, USA).

### 3.8 Image analysis

To assess the reliability of imaging and measurement, the procedure was repeated on a separate occasion on 24 surfaces (labial, buccal, lingual, and occlusal) for six teeth. The differences between first and second occasion measurements were assessed for each surface type by measurement of tooth surface area, hypoplastic lesion and opacity. Post-eruptive breakdown was not assessed as the measurement techniques used were identical to the other defect types. Fleiss’ intra-class correlation coefficient (ICCC)^29^ was calculated for opacity and hypoplastic area and tooth view surface area from the buccal, lingual and occlusal aspects (all tooth types combined). The Fleiss results were then categorised for the levels of agreement as described by Donner and Eliasziw (1987).^30^

## 4 Results

The Fleiss ICCC values showed excellent repeatability for the image analysis measurements (Table 1). Kappa values indicated good or excellent intra-operator repeatability and inter-operator reproducibility (Table 2). Hypoplastic pits on the occlusal surfaces were the most frequent defect in this sample (82.6%) (Table 3). The occlusal surface displayed far more post-eruptive breakdown than the other surfaces (39.13%) (Table 3) whilst the incisal portion of the buccal surfaces showed most diffuse opacities (53.4%) (Table 3). Image analysis methods showed the largest mean hypoplastic pit areas were on the lingual surfaces. The largest mean post-eruptive breakdown areas were on the lingual surfaces of posterior teeth. The largest mean demarcated opacity areas were found on the labial surfaces (Table 4).

The logistic regression analysis using the Hosmer and Lemeshow test for lack of fit was carried out on all findings and confirmed the suggested findings.^27^

The similarity measure found that the most commonly combined defects were post-eruptive breakdown and demarcated opacities.

## 5 Discussion

As both the EDI scoring and image analysis measurements were carried out on the digitally acquired images it was of great importance to provide optimum levels of standardisation whilst capturing these images. In particular the type, standardisation and manipulation of illumination, is of great importance in studying enamel defects. Fluorescent strip lights were used as the main source of illumination in this study. To eliminate reflections the teeth were also imaged using flashlights with polarising filters and this type of light was advantageous for quantifying opacities. However, it made the hypoplastic defects look shallow and indistinct.

For hypoplastic lesions a non-polarised normal translucent tooth appearance was advantageous as light and shadow provided definition of lesion boundaries. A method was developed, that captured the four images of each tooth surface used in this study, using four lighting conditions.

The teeth had been stored in alcohol, but during imaging in a pilot study it was noted that the teeth quickly dried out and the lesion outline appeared to change. Therefore in this study the teeth were transferred into saline and thymol for one week prior to imaging to re-hydrate. Excess moisture was dried using a soft tissue to reduce light reflection. This allowed a more detailed examination of the tooth surface because the nature and extent of any defects were easier to identify.^31^ The permitted imaging time of up to 3 min avoided drying out of the teeth, but if this limit was exceeded the tooth was re-immersed in saline and thymol for 24 h to re-hydrate. Most of the imaging time was for tooth positioning; the image acquisition process was then quick. The fluorescence lights did not radiate heat, which helped to keep the tooth hydrated.

Many studies of enamel defects to date have relied on direct observation scoring which increases the subjectivity of already subjective approaches. Whilst conventional photography produces either colour/black and white transparencies or prints, they may be subject to development and processing errors and variability.^10,32^ We overcame these problems by using digital image acquisition with standardised object placement, camera position and lighting so that the investigator could observe and evaluate the resultant reliably obtained image in a matter of seconds before saving.

The reliability ICCC results^29^ for the EDI methodology gave a very high degree of repeatability with values falling in the category of excellent.^30^ Kappa values also demonstrated good operator agreement ranging between 0.69 and 0.84 (Fig. 2), providing further evidence that the image analysis methodology is a sound approach for the quantification of demarcated opacities and hypoplastic lesions as well as the tooth surface area. These data compare favourably with earlier or alternate methodologies although statistics can vary between authors. Shaw and Murray reported that the Al-Alousi Index gave 72% reproducibility^4^ whilst Dummer et al reported that the original DDE Index gave 89% reproducibility.^33^ Clarkson et al found that the modified DDE was 87% reproducible^34^ and Horowitz et al that the Tooth Surface Index of Fluorosis provided 72% reproducibility.^6^ Wong et al reported that the DDE produced Kappa values of 0.81 for tooth surfaces, 0.95 for identifying demarcated opacities, 0.78 for diffuse and 0.82 for dental hyposplasia.^35^ Elcock et al. reported that the EDI provided comparable or improved reliability with Kappa values of 0.72 (general defect), 0.84 for identifying opacities, 0.79 for hypoplasia and additionally 0.66 for post-eruptive breakdown.^9^ The binary scoring system, however, provided for far quicker scoring.

EDI scoring from digitally acquired images reduces some of the systematic errors inherent in this type of index by ensuring different examiners are scoring exactly the same view. The system has the advantage of an additional patient imaging option such that standardised images for *in vivo* scoring are also available. The extended EDI scoring of the sub-categories has provided a key tool for defining lesion and opacity presentation complementing the objective area measurements the image analysis provides.

When considering the findings from this valuable sample the logistic regression revealed a significant occurrence of hypoplasia and post-eruptive breakdown occlusally. This may be due to the irregular morphology of the occlusal surfaces that may reflect more light on imaging, thus facilitating detection of small hypoplastic pits that otherwise may be missed. Significant post-eruptive breakdown on the occlusal surfaces of posterior teeth could be related to the greater masticatory forces present on areas that may already be hypomineralised. Results of this study indicated no significant differences between buccal and lingual surface defect frequency in contradiction with other studies finding the buccal surfaces more affected.^23,36^

In agreement with other studies the incisal portions of the teeth were more affected by all types of defects than the cervical halves. The post-eruptive breakdown was more apparent on the incisal portions of the teeth and this is probably due either to a higher degree of calcification in the cervical region or that this region is remote from the immediate effects of mastication reducing the chance of fracture to hypomineralised enamel. The posterior teeth had more post-eruptive breakdown and hypoplasia than anterior teeth in agreement with other studies.^23,36^

It has been suggested that ameloblasts responsible for thick enamel are more sensitive to various disorders than those associated with thin enamel so increased hypoplasia on posterior teeth may be explained by differences in enamel thickness.^37^ This may also be explained by the difference in formed enamel at birth with less formed for the posterior teeth.^38^

The similarity measure found that the most commonly combined defects were post-eruptive breakdown and demarcated opacities. There was approximately four times the chance of post-eruptive breakdown being present if there was a demarcated opacity. This is expected as a demarcated opacity represents an area of hypomineralisation that is vulnerable at certain sites to breakage after eruption.

The benefits of acquiring digital images are that they can be saved and stored indefinitely or sent electronically to remote sites for further investigation. This allows a greater number of comparisons to be made between different studies in future.

This study has demonstrated that the extended EDI and image analysis combined can produce reliable, more detailed and objective data through assessment of a greater number of variables. The images were calibrated and included a scale so data could be presented as actual mm^2^ as well as percentage of surface coverage. With the high definition of the images and possible magnification for analysis, hypoplasia could be further divided into the sub-categories easily (the authors note that as different viewing media may display images with a degree of variation, care should be taken that extremes of brightness and contrast be avoided and where possible the display settings be standardised and preferable from the same manufacturer).

The macroscopic results obtained here for these Amelogenesis Imperfecta teeth can be related to histological findings to investigate the microscopic features and aetiology of these defects. As the images are standardised the variation in whiteness and colour could also be considered.

## 6 Conclusions

The extended EDI and the standardised image acquisition and analysis system provided additional complementary measurements. The new EDI sub-types enabled more detailed scoring of enamel defects than previously possible and objective measurements of the defects were provided by the use of image analysis. This study demonstrated more detailed and reliable phenotyping of enamel defects than previously possible to enhance genotype/phenotype studies of enamel defects.

The imaging process overcame problems associated with conventional measurement techniques and provided a standardised system useful in comparative and longitudinal studies. Areas of demarcated opacities and well-defined hypoplastic lesions were measured accurately, but there were limitations in quantifying diffuse opacities and generalised pitting.

## Disclosures

*Competing interests:* None declared.

*Funding:* See acknowledgement.

## Figures and Tables

**Table 1:** Fleiss’ intra-class correlation coefficient for repeat measures for hypoplastic lesions and opacity surface area and tooth view surface area for 24 buccal, 24 lingual and 23 occlusal surfaces (tooth types combined) using image analysis.

Surface measured	Fleiss’ ICCC	Donner % Eliasziw classification of *R*
Hypoplastic area	0.99	Excellent
Opacity area	0.99	Excellent
Buccal surface area	0.99	Excellent
Lingual surface area	0.99	Excellent
Occlusal surface area	0.99	Excellent

**Table 2:** Unweighted and Weighted Kappa values for reliability measurement for intra- and inter-operator assessment of all surface defects on 24 buccal and 24 lingual surfaces.

Surface	Unweighted Kappa	Landis and Kock classification *R*	Weighted Kappa	Landis and Kock classification *R*
Buccal				
Intra-operator	0.84	Excellent	0.80	Substantial
Inter-operator	0.74	Substantial	0.78	Substantial

Lingual				
Intra-operator	0.80	Substantial	0.76	Substantial
Inter-operator	0.69	Substantial	0.73	Substantial

**Table 3:** Tooth surface frequency (%) of enamel defect sub-types found using the extended EDI.

	View				
	Buccal		Lingual		Occlusal
	116^a^		116^a^		23^a^
	Incisal^b^	Cervical^b^	Incisal^b^	Cervical^b^	
Hypoplasia					
Pits	47.4	21.5	44.3	24.3	82.6
Areas	7.4	0.0	4.3	2.6	4.3
Grooves	4.3	3.4	1.7	1.7	0.0

Opacity					
Demarcated	7.8	10.3	16.5	21.1	13.1
Diffuse	53.4	47.4	53.0	37.4	39.1

Post-eruptive breakdown	21.6	3.4	13.0	6.9	39.13

Percentages add to more than 100% as more than one defect was present on some surfaces.

a Number of teeth.

b Sites.

**Table 4:** Mean defect size per tooth surface type found using image analysis.

Mean total defect size (mm) and percentage of tooth surface with any defect			
	Labial (116)^a^	Lingual (116)^a^	Occlusal (23)^a^
Hypoplasia			
Pits	3.0	2.4	1.6
Areas	4.0	6.0	0.0
Grooves	2.2	0.0	0.0

Post-eruptive breakdown	3.9	5.5	3.2
Demarcated Opacity	2.8	2.6	0.8

a Number of teeth in parathesis.
